# Underserved communities in the radiation therapy land of plenty – Physicists’ perspective

**DOI:** 10.1002/acm2.14252

**Published:** 2024-01-04

**Authors:** Robert Praeder, Timothy Solberg, Afua A. Yorke

**Affiliations:** ^1^ Locum Physicist Ammon Idaho USA; ^2^ Department of Radiation Oncology University of Washington Seattle Washington USA

A phrase that's often used in the space of global health research is “global health is local health.” Yet in the space of local health, few publications and very little of the attention of professional organizations within our sphere, including the American Association of Physicists in Medicine's (AAPM) and the American Society for Radiation Oncology (ASTRO), have prioritized access to care in rural and underserved communities in the United States. While the AAPM has an entire international council focused on a myriad of global issues, there is nothing similar for underserved communities in the United States. ASTRO has the ARRO Global Health Subcommittee, and within the United States prioritizes access to care almost exclusively in the context of Medicare reform and prior authorization. To its credit, the Radiation Oncology Institute (ROI) recently launched a funding mechanism to address geographic barriers and “reduce disparities in radiation care for underserved populations in domestic or global settings.” In this editorial, we highlight the scope of the access to healthcare problem here in the United States, with a goal of raising awareness and activism within the clinical medical physics community.

Rural areas are one example of communities in the United States challenged with access to healthcare. Deaths from heart disease, cancer and stroke are all higher in rural areas, and the gap is widening, with studies suggesting the mortality gap between rural and urban areas has doubled since 2000.^1^ There are far fewer physicians per capita in rural areas than in non‐rural areas: one fifth of the US population resides in rural areas while only one tenth of all physicians practice in these areas.^2^, ^3^ Oncology specialists, as is the case with most medical specialties, are even more concentrated in urban areas; only 3% of medical oncologists practice in rural areas, and at the same time over 70% of counties in the United States have no access to medical oncologists.^2^ The 2017 ASTRO Workforce Study^4^ found that 88% of radiation oncologists practiced in urban or suburban areas (of whom 18% planned to retire in the next 5 years), and that only 13% practiced in the rural areas that account for roughly 20% of the US population (of whom 30% planned to retire in the next 5 years). On a macroscopic scale, the United States has largely modern technologies and the number of radiotherapy machines per million population is quite high compared to other Lower‐and‐Middle income countries (LMICs). However, on the granular level, 1 in 20 Americans live far from a radiotherapy clinic.^5^ Herb et al.^6^ found that 92.4% of US census tracts were within 1 h of the nearest radiation oncology facility, but that among rural tracts 34.4% were more than 1 h away from the nearest radiation facility, and that for 3054 isolated rural tracts the estimated travel time was 58 min longer than it was for urban tracts. The above cited travel times assume access to reliable transportation. According to a 2014 US Department of Agriculture study,^7^ over 1.6 million rural households do not have cars, with the highest proportion in the South, Appalachia, Southwest, and Alaska. Distances requiring a travel time of 2 h or more to the nearest radiation center are a major inconvenience even for those with reliable vehicular transportation. For those without a car, or who are unable to drive due to their medical condition, a distance of this magnitude to the nearest cancer center could present an insurmountable obstacle to receiving needed care.

Does the fact that there are fewer oncology specialists and treatment centers per capita in rural areas of the United States merely inconvenience cancer patients in these areas by requiring them to travel further and longer for treatment, or does having fewer oncology specialists and having to travel further to see them, additionally result in poorer treatment outcomes? While studies addressing this question have had differing conclusions, some have suggested that cancer treatment outcomes may be poorer for residents of rural areas in the United States than they are for residents of non‐rural areas. Rodgers et al. observed geographic disparities leading to significantly poorer survival in individuals with colorectal cancer.^8^ More recently, LaVigne et al.^9^ in a 2023 paper found that “hot zones” of all‐stage breast and prostate cancer death rates (defined as counties with death rates greater than or equal to 2 standard deviations above mean rates) were largely non‐metropolitan, tended to have a higher percentage of Black non‐Hispanic residents, and lower numbers of physicians per patient at risk. Markossian et al.^10^ found that women in small rural towns were less likely to undergo radiation for breast cancer than their urban counterparts and that women in higher poverty census tracts were least likely to undergo treatment. Aneja et al. found that when compared to counties with no radiation oncologists, counties with at least one radiation oncologist had statistically significant lower per capita mortality for colorectal, esophageal, prostate, and pancreatic cancer.^11^, ^12^, ^13^, ^14^

While the above cited papers establish correlation between low per capita radiation oncology resources and poorer cancer treatment outcomes, they do not establish cause and effect. Perhaps the critical factor in some cases could be lack of access to primary care providers leading to late detection of cancers. Nevertheless, we believe the correlations that have been found are troubling enough to make it important to pursue further investigations to rule out the possibility that lack of radiation oncology resources are leading to poor treatment outcomes in rural areas.

The papers by La Vigne et al.^9^ and Markossian et al.^10^ cited above are not the only ones to have found correlations between socioeconomic factors, race, and cancer treatment outcomes in addition to, or independent from, correlations between rural residence and outcomes. Jatoi et al.^15^ found that from 2014 to 2018 Black women were experiencing an approximate 40% higher breast cancer mortality risk compared with non‐Hispanic White women. McClelland et al.^16^ surveyed 23 studies examining differences in treatment outcomes and decisions between African American and non‐African American women. Most of these studies found that African American women were less likely to receive radiation therapy after breast conserving surgery. Among these studies a Surveillance, Epidemiology and End Results data (SEER) data study by Yeboa et al. of 67 124 women who underwent lumpectomy for stage I breast cancer found that African American Women were 18% less likely to receive RT after lumpectomy,^17^ and a SEER data study by Du Xianglin et al. of 89 110 stage I and II breast cancer patients found that African American Women were 24% less likely to receive adjuvant RT.^18^ Similar patterns have also been found for other disease sites. A SEER data study by Obirieze et al. of 54 400 low‐risk prostate cancer patients found that African American men were 42% less likely to receive prostatectomy or RT than Caucasian men and had higher all‐cause mortality than Caucasian men.^19^ Abdollah et al.^20^ found that Black men with metastatic prostate cancer were 26% less likely to receive radiation therapy in their last 12 months of life compared to their counterparts in other racial groups. A SEER data study by Simpson et al. of 11 216 stage IV colorectal patients aged 65 or older found that compared to Caucasian men, African American men were 30% less likely to receive RT and had 15% greater mortality,^21^ but that after adjustments for differences in treatment the differences in mortality were no longer statistically significant. McClelland et al. also surveyed disparities in care for American Indian, Hispanic, and Appalachian communities.^22^, ^23^, ^24^

A study by Voti et al. found that the probability of local breast carcinoma patients in Florida being treated with breast conserving surgery plus radiation (BCSR) instead of mastectomy for local breast carcinoma depended on their type of insurance, as well as on distance to the nearest radiation facility, race, age, and marital status. The percentage of patients receiving BCSR was 51% for patients insured by Medicaid, 53.3% for those without insurance, 56.5% for those on Medicare, and 64.3% for those with private insurance.^25^ What could account for poorer treatment outcomes for some socioeconomic or demographic groups, living in, or close to, urban areas with well‐funded cancer centers with the latest equipment? One of the authors believes they know why, from their personal experiences. In their first job out of medical physics training they spent most of their time doing 3D treatment planning at a 100 million dollar plus proton therapy center at an academic medical center, but also rotated to check charts at a county hospital several miles away (i.e., where the patients with bad or no insurance were sent), where patients were treated on a Cobalt machine based on 1D MU calculations or rarely, single slice 2D plans based on wire contours. The percentage of non‐white patients was much higher at the county hospital than it was at the medical school center with the proton treatment facility. At their next job in the outreach physics department at a medical school physics department heavily involved in IMRT research where many treatments used IMRT, they primarily covered a county hospital where, when they arrived, patients were being treated mostly with single slice wire contour 2D plans on a decades old single energy 6X machine with no RV. When they left 3 years later, the equipment had been upgraded to a CT sim and 2100CDex (with dual x‐ray energies, electrons, MLC and RV), and most plans were 3D, but IMRT was yet to be implemented.

Should those of us at well‐funded urban cancer centers with the latest and most expensive equipment be concerned if patients in our community with poor or no insurance or in US rural communities are being treated with equipment and techniques that we phased out 10 or 20 years ago? If so, should we be less, equally, or more concerned about this than with what care patients are receiving in low and medium income countries? Should the role of those employed at government funded medical schools be only to pursue ever more expensive and exotic new technologies for the flagship campus, or should their role also be to push those technologies out to communities who desperately need them? If not, whose role, if anyone's, should this be?

There is increasing evidence that there is shortage of Qualified Medical Radiation Therapy Physicists with the shortage being felt most severely at rural radiation centers. A recent publication^26^ in Physics Today, has sounded the alarm on the decline of radiation workforce and attributed this shortage to a surge in retirements. This shortage of the workforce poses a threat to advances in cancer therapy, diagnostic imaging, and the physiological impact of radiation. A recent discussion on a Reddit online forum revealed that many medical physicists shared sentiment that there is a shortage of medical physicists in smaller and more remote communities. Several such institutions reported taking 2 years to fill vacancies. One of the authors recently completed a 2‐year locum assignment at an institution in a rural state (city population 196 528 in a state with population of a population of 895 376) where it took that long to recruit a physicist to permanently fill an open position. At their previous locum assignment, again in a small city (population of 38 114 in a Metropolitan Statistical area with a population of 100 089) in a mostly rural state, the institution has been filling one of their two physics positions with a locum since October 2021 (2 years as of the date this is being written) due to inability to recruit a permanent physicist. The recruiter who sent this author on the most recent of these two jobs is of the opinion that for 2 years there has been a severe shortage of medical physicists, due in part to an insufficient number of residency program graduates to replace retiring physicists, with the problem being most severe for smaller and more remote cities. A second recruiter has had difficulty identifying sufficient locum individuals because they are staying longer on assignments than previously as centers are taking longer to fill positions than they used to. The average number of “career positions” advertised per month in the AAPM placement bulletin has increased dramatically from 2017 to 2023 (Figure 1), consistent with the market conditions reported by the recruiters referenced above. The authors experiences and recruiters’ market assessments are in agreement with a 2019 PhD thesis by Swanson,^27^ which predicted that the annual number of therapy residency graduates would be 25 to 60 short of job market requirements for 2020 to 2030 if the number of therapy residency graduates per year was not increased substantially above the current level of approximately 150 per year. This is in stark contrast with the conclusions of Newhauser et al.^28^ who found that as of 2022 “the size of the domestic medical physics workforce appears in balance with the nation's needs.” This may be the case overall nationally but is certainly not the case in underserved communities.

**FIGURE 1:**
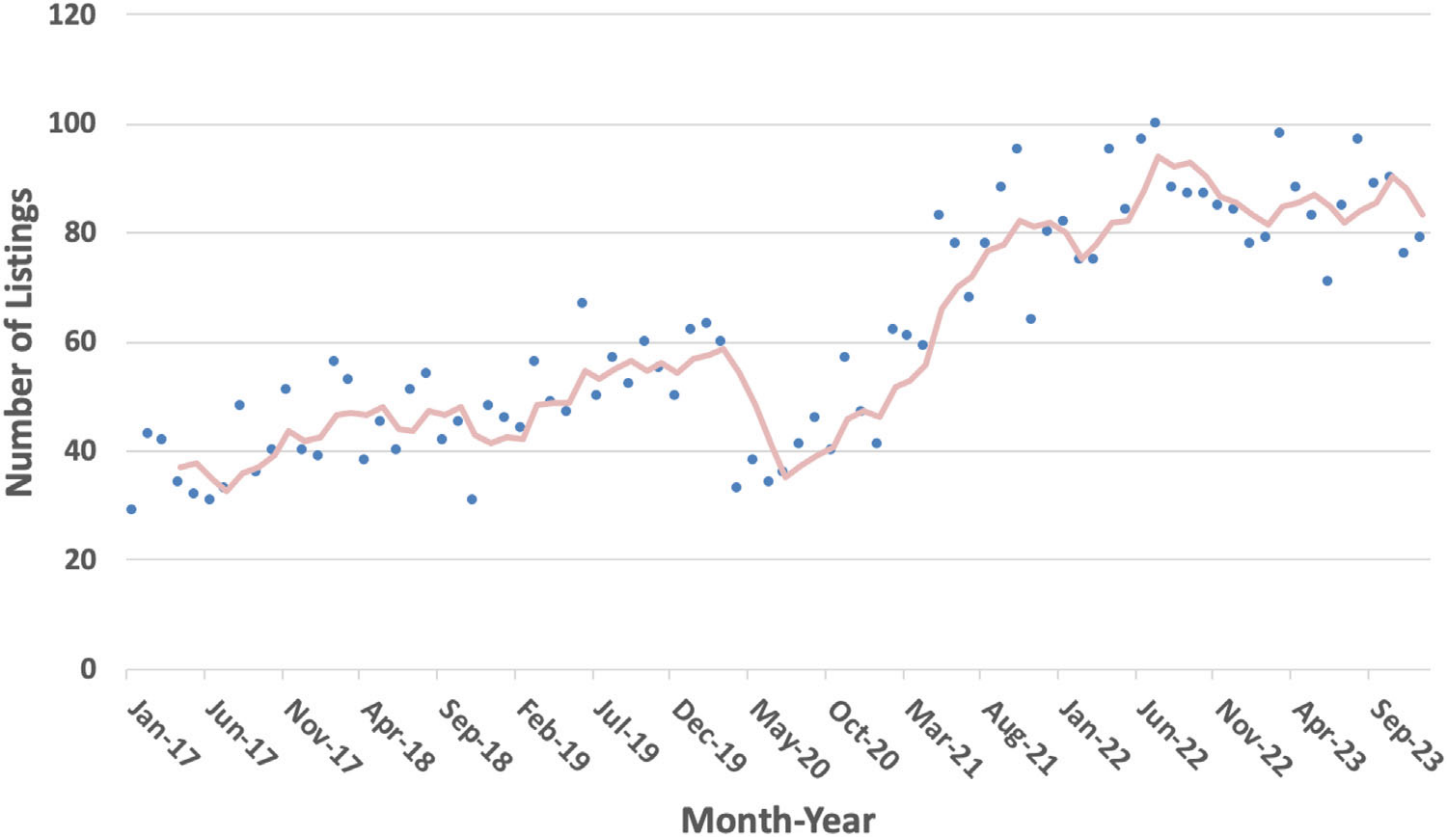
Average number of career positions per month (points) advertised on the AAPM website, from January, 2017 through December, 2023. Ads that were listed under the “CAMPEP Approved Residency” or “Training Positions” headings are excluded. The trendline shows a 4‐month moving average. A sharp drop due to the COVID pandemic is evident, followed by an accelerated rate post‐COVID. AAPM, American association of physicists in medicine's.

Is there enough evidence of a medical physicist shortage to warrant that the AAPM should be gathering data, if it is not already doing so, on how long it takes institutions in different regions and communities to fill positions? If such a survey finds that there is a shortage of medical physicists in some communities, should residency program positions be increased to fill these shortages? It is imperative to consider that as a professional organization we may run the risk that some centers may be perpetually unable to hire QMPs, and therefore re‐evaluate the duties that can only be performed by a QMP to allow for assistance by other individuals.

The CAMPEP “Standards for Accreditation of Residency Programs in Medical Physics” require that the number of program staff in a Residency Program must exceed the number of residents in a program by one.^29^ Thus, an institution must have at least three physicists to have a CAMPEP accredited residency program. The many rural clinics with only one or two physicists are thus unable to create their own residency program. In a recent discussion on the AAPM BBS, one physicist with experience setting up a residency program expressed the opinion that an institution typically needed to have four medical physicists to support a residency program. If this assessment is accurate then even rural clinics with three physicists could struggle to support a residency program. Perhaps alternative pathways be implemented to make it easier for institutions with one to three physicists to sponsor residency candidates for hub and spoke programs.

Should some fraction of residency recruitment focus on candidates from underserved areas who express interest in practicing in those areas, perhaps with such practice being encouraged by loan forgiveness after a minimum number of years of such practice? One partial model for such a program might be the WWMAI (Washington, Wyoming, Alaska, Montana, Idaho) Physician training consortium, a consortium between the University of Washington Medical School in Seattle and four predominantly rural states that have historically had physician shortages, and at the time of establishment of the program in 1970, did not have their own medical schools. Each member state, whose low population would have made it difficult for them to set up their own medical school, sponsors a set number of seats at the University of Washington Medical School for residents of their states.^22^ The rural member states subsidies of these seats are forgiven if graduates of the program practice for a minimum number of years in their home states.^30^ The first year of basic science training is in the home state, second year clinical training is at the U.W. Medical school in Seattle, and the third and fourth‐year training is at clinical sites across all five WWAMI states. The needs of four rural states with physician shortages who had no medical schools, have thus been addressed at much less expense than creating a new medical school in each state.

It is important that as medical physicists, our focus on global health does not sideline our responsibility regarding access to care in rural America and underserved populations in the United States. If Global health is truly local health, then our focus cannot just be international. It is time we paid equal attention to communities in the United States where shortage of radiation oncology staff, equipment, and restricted access to care affects patient outcomes.

To be an organization that puts patients first is to come to the table to identify the causes of poorer health outcomes for all communities in the United States and devote time and effort to addressing any issues found that are within our scope of practice and influence that are contributing poor patient outcomes in these communities. This includes acting promptly to identify shortages of qualified medical physicists in all communities and acting promptly and effectively to address the issue.

The scope of influence for individual staff physicists may be very limited, but activism most always starts at an individual level. And for those in leadership positions, such as medical school department chairs, and physics directors, perhaps their scope of influence could expand to include things such as establish outreach physics programs, develop accessible short courses in new technologies for community physicists, or partner with their school's public health department to investigate patterns in cancer treatment outcomes across their states. Physics residency program directors could pursue training rotation relationships with smaller community institutions, or allowing institutions that are too small to have residency programs to sponsor locally recruited candidates for their residency programs. State and federal governments could expand sources of funding for residency programs in underserved areas (as in WWAMI) or to organizations who are otherwise unable to establish their own residency program (e.g., regional hospital and cancer center, physics consulting groups, and other government organizations or NGOs with an interest in increasing health care access to underserved communities). And for those in AAPM leadership, expand your scope of influence to include underserved communities in the United States in addition to existing programs that focus on low‐ and medium‐income countries. The travel may not be as exotic, but the impact can be profound.

## AUTHOR CONTRIBUTIONS

All authors reported on this publicaton contributed to the preparation of this manuscript.

## CONFLICT OF INTEREST STATEMENT

The authors declare no conflicts of interest.
